# A qualitative systematic review of factors influencing parents’ vaccination decision-making in the United Kingdom

**DOI:** 10.1016/j.ssmph.2016.07.005

**Published:** 2016-08-30

**Authors:** Alice S. Forster, Lauren Rockliffe, Amanda J. Chorley, Laura A.V. Marlow, Helen Bedford, Samuel G. Smith, Jo Waller

**Affiliations:** a Health Behaviour Research Centre, UCL, Gower Street, London WC1E 6BT, United Kingdom; b Institute of Child Health, UCL, 30 Guilford Street, London WC1N 1EH, United Kingdom; c Wolfson Institute of Preventive Medicine, Queen Mary University of London, Charterhouse Square, London EC1M 6BQ, United Kingdom

**Keywords:** Thematic synthesis, Vaccination, Parents, Patient Acceptance of Health Care

## Abstract

**Background:**

High uptake of vaccinations is crucial for disease prevention. Although overall uptake of childhood immunisations is high in the United Kingdom (UK), pockets of lower uptake remain. Novel systematic methods have not been employed when reviewing the qualitative literature examining parents’ vaccination decisions.

**Aims:**

We aimed to conduct a qualitative systematic review of studies in the UK to understand factors influencing parental decisions to vaccinate a child.

**Methods:**

On 12/2/14 we searched PsycINFO, MEDLINE, CINAHL plus, Embase, Social Policy and Practice and Web of Science for studies using qualitative methods and reporting reasons why parents in the UK had or had not immunised their child. Participant quotes and authors’ interpretations of qualitative data were extracted from the results of articles. Thematic synthesis was used to develop higher-order themes (conducted in 2015).

**Results:**

34 papers were included. Two types of decision-making had been adopted: non-deliberative and deliberative. With non-deliberative decisions parents felt they had no choice, were happy to comply and/or relied on social norms. Deliberative decisions involved weighing up the risks and benefits, considering others’ advice/experiences and social judgement. Emotions affected deliberative decision-making. Trust in information and vaccine stakeholders was integral to all decision-making. Practical issues affected those who intended to vaccinate.

**Conclusions:**

Parents adopted two different approaches to decision-making about childhood vaccinations. By understanding more about the mechanisms underpinning parents’ vaccination behaviour, in collaboration with vaccine stakeholders, we can better design interventions to enhance informed uptake.

## Highlights

• This qualitative systematic review identified 2 types of vaccine decision-making.
• Some parents’ decision-making was non-deliberative, while others’ was deliberative.
• Findings will help design interventions to enhanced informed uptake of vaccines.

## 1 Introduction

Vaccination is a vital public health intervention for the prevention of communicable diseases. Its effectiveness has been demonstrated by the eradication of smallpox, the near eradication of poliomyelitis and significant reductions in the incidence of vaccine preventable diseases (WHO, 2016a, 2016b). High uptake is crucial to the success of vaccination programmes and if a sufficient proportion of a population are vaccinated, protection is also provided to those who have not been vaccinated (herd immunity). In the United Kingdom (UK), uptake of recommended childhood vaccinations is high (Public Health England, 2014, Public Health England, 2015), however disease outbreaks have occurred where pockets of susceptibility remain (Public Health Wales, 2015).

Under most circumstances, UK parents are required to provide consent for children under the age of 16 to receive vaccinations (although individuals <16 years can provide consent if they are deemed competent to do so) (Public Health England, 2015). Understanding why parents do or do not accept vaccinations is complex. Some parents may unquestioningly accept or reject vaccinations, while others experience uncertainty, which may delay or result in rejection of immunisation and some experience barriers that prevent immunisation (Samad et al., 2006, Larson et al., 2014, MacDonald, 2015, Robison et al., 2012).

There is a pressing need for the development of interventions to address sub-optimal vaccination uptake among those experiencing uncertainty about vaccines (Marlow et al., 2008, Gordon et al., 2011, Bosch et al., 2012, Franco et al., 2012, NICE. Reducing differences in the uptake of immunisations (PH21), 2013). Behavioural medicine has afforded researchers with the tools to develop effective interventions, but to do so it is important to understand the determinants of vaccination uptake. This is best achieved by rigorously reviewing the existing literature, much of which in this field has been qualitative (providing a rich and in-depth picture of the research area).

While qualitative systematic reviews have been published that explore the determinants of vaccination uptake, novel approaches to systematically synthesising qualitative data have not been adopted (to our knowledge one review has used such techniques to synthesise data pertaining to HPV vaccination (Ferrer, Trotter, Hickman, & Audrey, 2014) and one pertaining to combination vaccines (Brown, Kroll, & Hudson, 2010)). While traditional systematic reviews aim to collate and summarise existing knowledge, methods for synthesising qualitative literature attempt to go beyond simple aggregation. Through comparison across studies and conceptual interpretation, methods for qualitative synthesis seek to generate a new and fuller understanding of the phenomenon of interest, while maintaining rigorous and transparent methods and standards (Barnett-Page and Thomas, 2009, Gough et al., 2012, Jensen and Allen, 1996, Thorne et al., 2004). Parents’ vaccination decisions are context-specific (MacDonald, 2015), so any exploration of these decisions needs to be done by country, although the decision-making processes are likely to have commonalities across contexts and findings can be extrapolated to other similar countries. We present findings of a qualitative systematic review that aimed to understand the factors influencing UK parents’ decisions to vaccinate a child.

## 2 Materials and methods

We conducted a systematic review of qualitative studies exploring factors that influence parents’ decisions to vaccinate a child as part of the UK childhood immunisation programme (NHS Choices, 2016). On 12/2/14 we comprehensively searched PsycINFO, MEDLINE (Ovid version of PubMed), CINAHL plus, Embase, Social Policy and Practice and Web of Science for studies conducted in the UK at any time, examining vaccination and using qualitative methods (see Supplementary Material for search terms and inclusion/exclusion criteria). Reference lists of included articles were searched for relevant articles and citation searching was performed using Web of Science.

Articles were included if they reported qualitative findings (e.g. from interviews, focus groups, free-text survey responses) and were published at any time in peer reviewed journals in English. We excluded letters, dissertation abstracts, book chapters, reviews and commentaries. Outcome data (quotes that had been reported and author interpretation of qualitative data) were extracted from the results sections of articles/abstracts.

After duplicates were removed, titles were reviewed by AF to exclude articles that obviously did not meet inclusion criteria. All abstracts and then full text articles were reviewed by AF, LR, AC and SS. ‘Excluded’ articles were checked by another researcher and disagreements resolved by discussion.

Thematic synthesis was used to identify important and recurrent themes (conducted in 2015) (Thomas & Harden, 2008). This method was developed based on the qualitative analytical technique ‘thematic analysis’ and borrows from traditional systematic review methods. It was developed with the aim that the findings of reviews using the method should be usable and accessible to policy makers and researchers, and could be used to develop interventions. Firstly AF, LR and AC coded one third of the text each, line-by-line and developed descriptive themes following discussion. These were applied to the data by AF, LR and AC. Finally, analytical themes were generated by discussing the descriptive themes at length (AF, LR, AC, LM and JW) until consensus on interpretation was reached. Analysis was conducted using NVIVO (QSR International Pty, 2012). Study quality was assessed using the CASP tool (Critical Appraisal Skills Programme, 2013). Studies with scores of 0-4 were high risk of bias, and 5-9 low risk. Findings are reported following PRISMA (Supplementary Material) and ENTREQ guidelines (Moher, Liberati, Tetzlaff, Altman, & Group, 2009; Tong, Flemming, McInnes, Oliver, & Craig, 2012).

## 3 Results

**Fig. 1:**
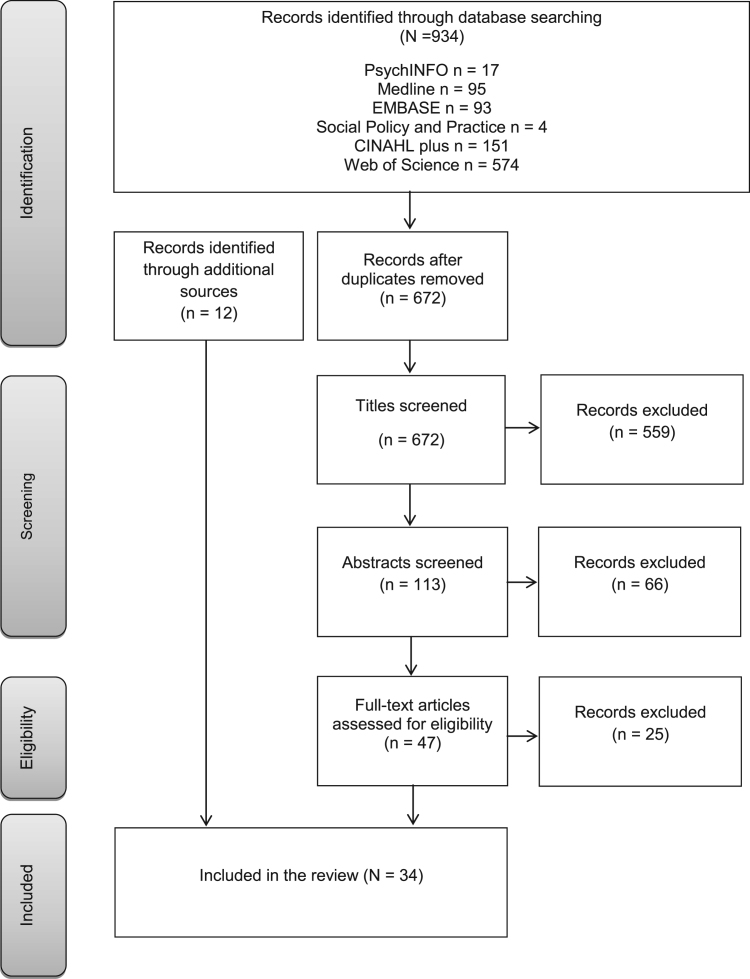
Flow diagram of included studies, adapted from Moher et al. (2009).

The search identified 934 articles. Excluding duplicates (*n*=262), 672 titles were assessed. There were 559 articles excluded based on their title, 66 based on their abstract and 25 after reviewing the full text. Hand searching reference lists and citation searching identified an additional 12 articles. In total 34 articles were included (Fig. 1, Table 1, Supplementary Material), published between 1994 and 2014 and comprising a total of 1,997 participants (range: 5–950). Most (>91%) participants were mothers. The majority of articles focused on MMR (*n*=17) or immunisation in general (*n*=11) (HPV 5, influenza 1, DTaP/IPV/Hib 1). Most used interviews (*n*=18) or focus groups (*n*=9) (free text questionnaire responses 3, participant observation 1). Where described, data were frequently analysed using thematic analysis (*n*=7), grounded theory (*n*=6), constant comparison (*n*=6) or framework analysis (*n*=3) techniques (5 other articles each used a different analytic technique). Thirty articles were low risk of bias and four high risk of bias.

**Table 1:** Characteristics of included studies.

**Lead author**	**Aim**	**Population of interest**	**Participants**	**Data collection period**	**Study design**	**Analysis**	**Vaccination of interest**	**Risk of bias**
Anderson (2002)	To study the context of child care decision making by inner city and suburban mothers	Inner city and suburban mothers of new-borns	131; Female	Not described	Free text questionnaire responses	Not detailed but analytical process described	Childhood vaccination in general	High
Austin (2001)	To understand parents' experiences of deciding to have their child immunised	Parents of children aged 7-18 months who had recently been immunised	13; Male (2) and Female (11)	Not described	Semi-structured interviews	Staged process	MMR (other vaccines considered that are not part of UK programme)	Low
Austin (2008)	To hear parents' stories about immunising their children and to compare the views of parents of completely and incompletely immunised children	Parents of children aged 5-6 years	25; Male (1) and Female (24)	Not described	Focus groups	Spiral analysis	Childhood vaccination in general	Low
Brown (2012)	To explore parents’ MMR decision-making	Mothers planning to accept, postpone or decline the first MMR dose for their 11-36 month old children	24; Female	June 2008 to March 2009	Semi-structured interviews	Modified grounded theory	MMR	Low

Brownlie (2005)	To explore the role of trust in parents’ vaccination decisions	Parents from different deprivation backgrounds and with children (aged 2-18 months) of different MMR invitation stages (pre/post)	85; Male (7) and Female (78)	1998 and 2001	Focus groups	Not described	MMR	Low
Casiday (2007)	To explore parents' decision-making about the MMR vaccination	Parents of young UK children	87; Male (10) and Female (77)	November 2002 to October 2004	Focus groups and interviews	Not detailed but analytical process described	MMR	Low
Condon (2002)	To explore attitudes of ethnic minority parents to preschool immunisations, particularly first MMR vaccination	Mothers of children aged 16 months to 3 years of Pakistani, Somali and African-Caribbean ethnicity	21; Female	November 2000 to March 2001	Semi-structured interviews and focus groups	Thematic analysis	Childhood vaccination in general	Low
Cunninghame (1994)	To survey reasons for non-uptake and attitudes to immunisation and immunisation services within the orthodox Jewish community in London, UK	Parents from the orthodox Jewish community	67; Gender not described	June 1991 to March 1992	Interviews	Not described	Childhood vaccination in general	High

Evans (2001)	To investigate what influences parents' decision-making regarding MMR immunisation and the impact of the controversy over its safety	Parents with children aged between 14 months and 3 years of age	48; Male (5) and Female (43)	Not described	Focus groups	Modified grounded theory and constant comparison	MMR	Low
Gardner (2010)	To extract underlying beliefs towards MMR vaccination from UK parents' views towards potential motivational and organisational interventions	Parents living in London	28; Male (8) and Female (20)	Summer 2008	Focus groups	Thematic analysis	MMR	Low
Gordon et al., (2011)	To explore attitudes to human papillomavirus (HPV) vaccination and reasons for accepting or declining the vaccine in the British Jewish community	Jewish mothers of girls offered the HPV vaccination	20; Female	June 2010 to September 2010	Interviews	Framework analysis	HPV	Low
Guillaume (2004)	To examine the MMR vaccination scare, its impact on parents of young children, and its effect on their need for information	Parents of children <5 years of age	17; Male (1) and Female (16)	February 2002	Semi-structured interviews	Not detailed but analytical process described	MMR	Low

Henderson (2008)	To assess reasons for low uptake of immunisation among orthodox Jewish families	Mothers from the orthodox Jewish community	25; Female	May 2003	Interviews	Modified grounded theory	MMR (and two vaccines not included in UK programme)	Low
Henderson (2011)	To explore parents' and girls' understanding of the protection offered by the HPV vaccine, or the need for future screening	Parents of 12 and 13 year old girls who had been offered HPV vaccination	26; Male and Female	July 2009 to June 2010	Semi-structured interviews	Thematic analysis and constant comparison	HPV	Low
Hill (2013)	To ascertain factors influencing parental immunisation decision making	Parents of children who have received the MMR vaccination	5; Male (1) and Female (4)	July 2010	Semi-structured interviews	Modified grounded theory	MMR	Low
Hilton (2006a)	To examine how British parents conceptualise the notion of ‘immune-overload’ and how they relate this concept to their own children	Parents with a range of ages, socio-economic circumstances, and family circumstances	72; Male (8) and Female (64)	November 2002 to March 2003	Focus groups	Constant comparison	Childhood vaccination in general	Low
Hilton (2006b)	To explore parents' understandings of the diseases included in the current UK Childhood Immunisation Programme	Parents with a range of ages, socio-economic circumstances, and family circumstances	72; Male (8) and Female (64)	November 2002 to March 2003	Focus groups	Constant comparison	Childhood vaccination in general	Low

Hilton (2007)	To examine parents' views on the role the media, politicians and health professionals have played in providing credible evidence about MMR safety	Parents with a range of ages, socio-economic circumstances, and family circumstances	72; Male (8) and Female (64)	November 2002 to March 2003	Focus groups	Constant comparison	MMR	Low
Johnson (2014)	To explore mothers’ engagement with advice around the combined MMR vaccine	Mothers of children aged 12-18 months	5; Female	2011	Focus group	Thematic analysis	MMR	Low
Kennedy (2014)	To explore vaccination views in Scotland among parents across three vaccines	Mothers resident in Scotland	15; Female	2008 to 2010	Interviews	Thematic analysis	MMR and HPV (also H1N1, not included in review)	Low
Lewendon (2002)	To identify local factors contributing to poor immunisation uptake	Parents in areas of low vaccine uptake in South Devon, UK	Not described	1998 to 1999	Focus groups	Not described	Childhood vaccination in general	High
Marlow (2009a)	To assess HPV awareness and HPV vaccine acceptability in a sample of women representing the major UK ethnic minority groups	Women from various ethnic backgrounds	950; Female	July 2008 to August 2008	Structured questionnaire interviews	Not detailed but analytical process described	HPV	Low

Marlow (2009b)	To explore attitudes to HPV vaccination among Black and Asian mothers living in Britain	Black and Asian mothers living in Britain	20; Female	April 2008 to August 2008	Interviews	Framework analysis	HPV	Low
McMurray (2004)	To explore parents’ accounts of decision making relating to the MMR vaccine controversy, identifying uptake determinants and education needs	Parents with children <1 year of age	69; Male (4) and Female (65)	January 2002 to June 2003	Semi-structured interviews	Framework analysis	MMR	Low
Mixer (2007)	To explore ethnic differences in knowledge, attitudes and behaviour related to immunisation	Mothers from various ethnic backgrounds	37; Female	Not described	Focus groups	Thematic analysis	MMR	Low
Petts (2004)	To describe the information strategies that parents use to make sense of health risk issues	Parents of children with various MMR vaccination status'	64; Male and Female	February 2002 to July 2002	Focus groups	Analytic deduction	MMR	Low
Poltorak (2005)	To explore how mothers think and decide about MMR vaccination for their infants.	Mothers with children <3 years of age.	23; Female	Not described	Group discussions, participant observations and in-depth narrative interviews	Not detailed but analytical process described	MMR	High

Raithatha (2003)	To assess vaccine risk perception among parents who have their children immunised	Parents of nursery aged children	15; Male (1) and Female (14)	Not described	In-depth interviews	Interpretive phenomological analysis	Childhood vaccination in general	Low
Sampson (2011)	To explore parental reasons for non-uptake of influenza vaccination in young at-risk groups	Parents of children identified as being at risk for influenza but who had not received vaccination	16; Gender not described	November 2008	Interviews and free text responses from questionnaires	Not detailed but analytical process described	Influenza	Low
Smailbegovic (2003)	To explore the knowledge, attitudes and concerns regarding immunisation and vaccine-preventable infections in parents whose children have not completed the recommended course	Parents of children resident in London, UK	Female (10)	Not described	Interviews	Not described	Childhood vaccination in general	Low
Sporton (2001)	To explore the decision-making process of parents who have chosen not to have their children immunised	Parents of children aged between 7.5 months and 20 years	13; Male (1) and Female (12)	Not described	Semi-structured interviews	Consistent and systematic review	Childhood vaccination in general	Low

Tickner (2007)	To explore parental decision-making about the DTaP/IPV/Hib ‘five-in-one’ vaccine	Parents of children aged 4-13 weeks	22; Male (1) and Female (21)	November 2005 to November 2006	Semi-structured interviews	Modified grounded theory	‘Five-in-one’ Dtap/IPV/Hib	Low
Tickner (2010)	To explore parents’ views about pre-school immunization and to identify reasons for lower pre-school uptake compared with the primary course	Parents of children aged 2-5 years	21; Male (2) and Female (19)	April 2006 to December 2006	Semi-structured interviews	Modified grounded theory	MMR (and another vaccine no longer offered in UK)	Low
Tomlinson (2013)	To explore the health beliefs of Somali women resident in the UK	Somali women resident in the UK with one child <5 years of age	23; Female	February 2012 to April 2012	Semi-structured interviews	Thematic analysis	Childhood vaccination in general	Low

### 3.1 Overview

**Fig. 2:**
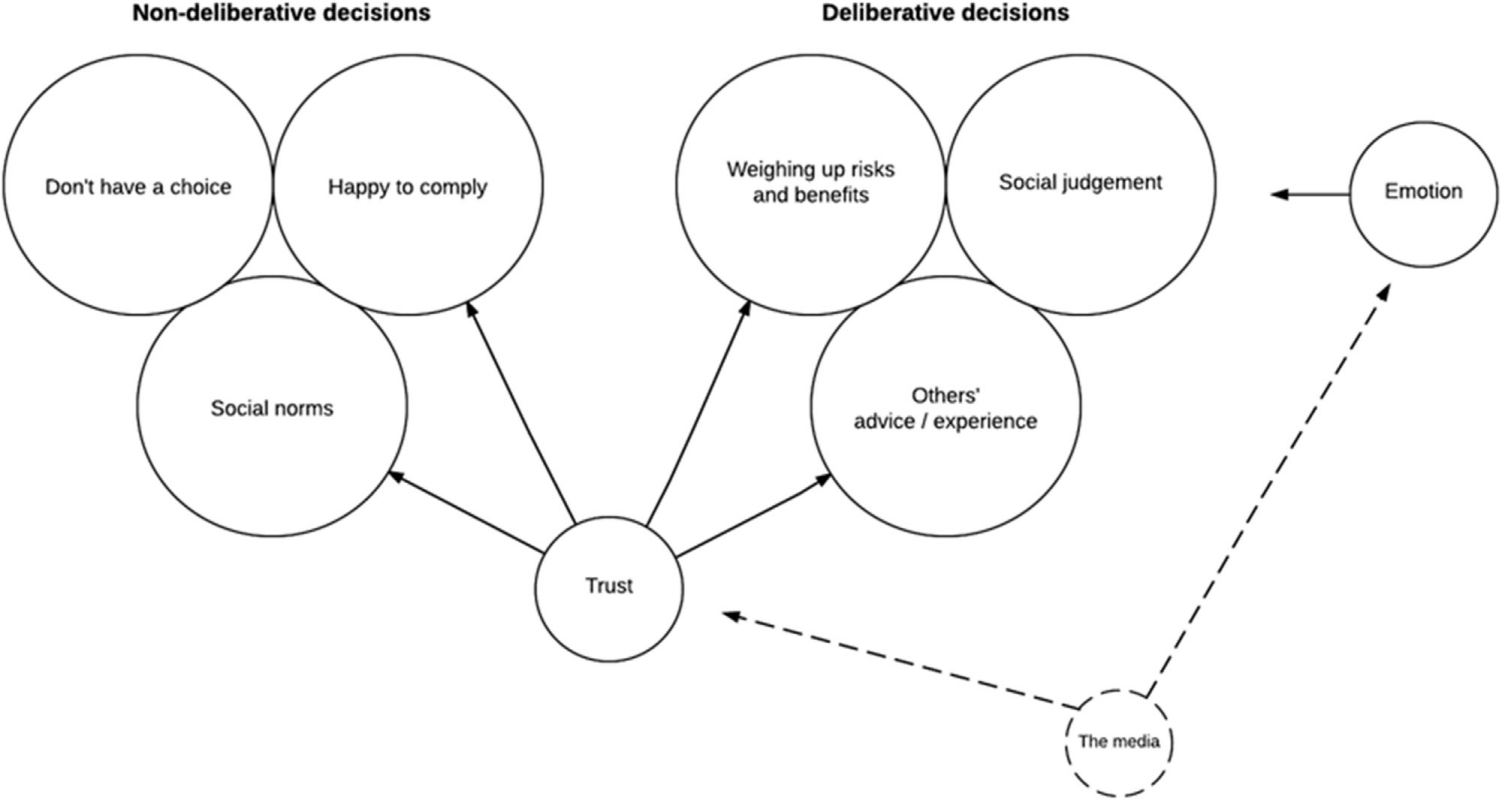
Themes identified and relationships between themes.

The thematic synthesis identified two types of decision-making used by parents: non-deliberative and deliberative (Fig. 2). These two approaches were not mutually exclusive and there was evidence that some parents adopted both approaches at different times. Non-deliberative decisions were those in which parents were happy to comply (theme 1), where parents did not think they had a choice (theme 2) and/or relied on social norms (copying others’ behaviour) (theme 3). Deliberative decisions involved parents weighing up the risks and benefits of vaccinating (theme 4), making an assessment of the appropriateness of vaccinating their child based on others’ advice/experiences (theme 5) and social judgement (feeling responsible and fearing judgement by others) (theme 6). Parents’ emotions (theme 7) affected the themes within deliberative decision-making, and the media sometimes influenced this. Trust (theme 8) (in information and vaccine stakeholders) was affected by the media and influenced Themes 2–5. Finally, (regardless of whether decisions were non-deliberative or deliberative) practical issues influenced whether those who intended to vaccinate their children actually did so (theme 9). Quotes are presented within the text (with first author name and whether it is an author/participant comment). Additional quotes are provided in Supplementary Material.

### 3.2 Non-deliberative decision-making

Most articles suggested parents spent much time considering their immunisation decisions; however, some made non-deliberative decisions.

#### 3.2.1 Theme 1: Compliant

For some, vaccination was seen as routine and this was positive. However, for others, vaccinating their child was an act of compliance, although not necessarily perceived as undesirable. Parents described being ‘guided’ to immunise and had accepted that complying with recommendations was appropriate.

“Immunisation… was something you were prompted to do by the system as part of the routine of having a baby, and you don't really think about” Johnson, author comment.

#### 3.2.2 Theme 2: Don't have a choice

Parents described feeling that they were under pressure to immunise, sometimes specifically mentioning that they felt they had no choice (including incorrectly believing that vaccination was a mandatory requirement for school-entry and fear of being removed from GP patient lists).

“I think you just feel pressurized anyway by health visitor and doctors: ‘this is the thing to do, we are doctors, we know what's best'” Marlow, participant comment.

#### 3.2.3 Theme 3: Social norms

Social norms were used by parents as a heuristic (cognitive shortcut) for their decision-making. Parents rationalised their decision because others they knew also did or did not vaccinate or it was not the ‘done’ thing in their culture. Some parents suggested that they did not do research before making a decision because they felt other parents had done this for them.

“Women's risk perceptions were largely influenced by their cultural norms and these made an important contribution to their decision not to accept HPV vaccination when it was offered to them” Gordon, author comment.

### 3.3 Deliberative decision-making

#### 3.3.1 Theme 4: Weighing up the risks and benefits of vaccination

One aspect of deliberative decision-making was weighing up the risks and benefits of vaccination, balancing the risks of contracting the disease, the severity of the disease, the effectiveness of vaccines and the risk of side-effects (Supplementary Material). This theme has been discussed extensively in the vaccination literature, so findings are summarised and presented fully in Supplementary Material. For most parents, vaccination decisions were a balancing act, however, some felt that no level of risk was acceptable.

“Although it might be a very, very small percentage risk, it's your child and if it gets that, you have to deal with that for the rest of your life” Brownlie, participant comment.

Parents considered whether vaccination was necessary to prevent the disease in question, based on their assessments of the severity of the disease (sometimes in relation to other diseases or the child's sex) and whether the child would be exposed to the disease. One parent explained, “I suppose because I was at home with him, for the first… year of life, I knew that he wouldn't be exposed to anything, he wasn't going to a nursery or a child minder… I knew that to some extent I had some degree of control over the people he was exposed to and the germs he was exposed to” (Sporton, participant comment). Many diseases were perceived not to be a particular threat in the UK. Some parents believed their lifestyles/environment protected their child sufficiently without the need for vaccination or alternatively provided reason to immunise. One mother explained “coming from a Muslim background… we don't have sex before marriage … because of that reason I'd probably say no…” (Marlow, participant comment about human papillomavirus (HPV) vaccination).

Knowledge of scientific reports, historical changes in disease prevalence, or a general trust in medicine informed parents’ assessments of whether vaccines are an effective way to prevent disease. Some parents held models of how the immune system works that were inconsistent with the current medical model of immunology and for others their beliefs in God or fate influenced their perceptions of vaccine efficacy.

“If children get measles, mumps, and rubella it helps build up their natural immunity, and that's better than the immunity built up by vaccines” McMurray, participant comment.

Parents carefully considered potential side-effects of a vaccine. Concerns about the safety of particular vaccines were either extrapolated to other vaccines or caused parents to perceive that some were lower risk than others.

“I've never heard anything adverse about the five-in-one …, not like MMR is constantly in the press. I never really hear about the five-in-one being bad, so erm I don't have an issue” Tickner, participant comment.

Concern that vaccinations might cause side-effects made parents assess the level of risk to their own child, considering family history and their child's history of illness or premature birth.

“...the second one had lots of colds, he had allergies and eczema, and em, it just seemed to be too much on his wee [little] immune system and I just felt it was too risky, whereas the third one is a much more robust child…” Hilton, participant comment.

Parents conceptualised the mechanisms by which vaccines cause harm in three ways: (1) by weakening the immune system or sending it into ‘over-drive’; (2) vaccine ingredients causing harm; and (3) vaccines causing an increase in high-risk behaviour (relevant for viruses with a ‘behavioural’ mode of transmission such as HPV).

#### 3.3.2 Theme 5: Others′ experiences and advice

Others′ experiences shaped parents′ vaccination decisions. Knowing other families who had positive vaccination experiences encouraged parents to accept vaccination for their own child. Some knew others who had negative experiences of the disease that vaccination was aiming to prevent, which raised their perceptions of their own child's vulnerability.

“Debbie… recounted how two of her friends had young sons who had MMR and were ‘fine’” Petts, author and participant comment.

Conversely, some parents had been influenced by others’ experiences of vaccine side-effects, which in some instances were considered severe. Although this did not always result in parents deciding not to vaccinate their child, it caused anxiety. Specific to MMR vaccine, parents who knew of children with autism were dissuaded from vaccination, presumably through fear of their child developing the condition.

“…a bloke I work with, his brother had it and his brother has got autism. He swears it was something to do with it” Petts, participant comment.

When parents knew of children who had not been vaccinated but remained healthy, they sometimes perceived their own child as being less vulnerable to that disease and less in need of vaccination. Similarly, parents who knew of others who had experienced the disease that the vaccine was aiming to prevent, but who had not suffered long-term side-effects, did not perceive the disease to be severe.

“...no parents mentioned that they knew of anyone who had suffered long-term damage [of contracting measles]. Indeed, their experiences of measles often rendered it a less threatening disease” Hilton, author comment.

Others′ advice influenced parents’ vaccination decision-making, including their families, particularly their mothers, as well as friends with older children.

“One described how her mother said ‘Oh you know, whooping cough is not so bad, you had whooping cough, you know. If there's any risk with the injections, don't get it because whooping cough's fine” Hilton, author and participant comment.

#### 3.3.3 Theme 6: Social judgement

Regardless of parents' decisions, many reasoned that their choice was part of being a ‘good parent’. Parents were sometimes aware that others (parents or health professionals) would judge them according to this principle, and themselves judged others who made decisions opposite to their own. Further pressure to accept vaccination was created through discourses of the social responsibility to contribute to herd immunity. Parents often mentioned this as secondary to protecting their child, but protecting the community was also reported as influencing the decision to vaccinate. Non-immunising parents used a second discourse of being a good parent, placing the wellbeing of their child above others to mitigate social pressures.

“My own children's health and safety is more important than the impact on the population” Casiday, participant comment.

Relatedly, in the context of the HPV vaccine (which protects against a sexually transmitted infection) parents reasoned that vaccination could invoke social judgement and preferred their child to remain unvaccinated over being stigmatised. One mother discussing the HPV vaccine stated that she did not feel a social responsibility to contribute to herd immunity because HPV is only transmitted through skin-to-skin contact.

#### 3.3.4 Theme 7: Emotions affecting decision-making

The role of others’ advice/experiences, social judgement and weighing up the risks and benefits were all influenced by emotional responses that affected decision-making. Emotions were only related to the act of making a deliberative decision. The media triggered emotional responses, particularly regarding side-effects. Fear, worry and guilt surrounding vaccination led some parents to decide against it or to defer the decision, whereas it motivated others to vaccinate. Parents described anticipating that they would regret vaccinating, while others anticipated regretting not vaccinating and some felt torn between the two.

#### 3.3.5 Theme 8: Trust in vaccine information and stakeholders affects non-deliberative and deliberative decisions

Parents discussed the issue of trust in relation to various key stakeholders and the information they provide. Trust was crucial to whether parents were happy to comply (theme 1) and whether to act in accordance with social norms (theme 3), or how parents interpreted the ‘evidence’ of the risks and benefits (theme 4) and valued others’ advice/experience (theme 5). As with Theme 4, this theme has been discussed extensively in the vaccination literature, so findings are summarised and presented fully in Supplementary Material.

Parents’ distrust in the government originated from historic health scares that remained in their memories, believing that the government conceals information. One author stated that “generally parents did not have confidence in statements issued by the government about the safety of MMR and analogies were made with the BSE [Bovine Spongiform Encephalopathy] crisis” (Evans, author comment). There was a perception that the government's motive for promoting vaccination was a cost-saving activity. Parents who distrusted vaccination research and drug development saw their children as being used as “guinea pigs” and disliked the uncertainty of scientific research.

“I think, well how can they just say that and just, so confidently, you know, think the atom is the smallest thing until they split it open and then it's not and they can just so quickly just change and I think that's, that's hard when you're trusting these people with your child's health” Johnson, participant comment.

Parents’ distrust in healthcare professionals was mainly based on the concern that GPs are financially rewarded for vaccine uptake, with one parent expressing “you're meant to trust your doctor implicitly and yet… they're getting paid for having so many people vaccinated…, and you start thinking ‘well… who's got my wee [little] boy's best interests at heart?” (Hilton, participant comment). Issues arising in the GP consultation, including rushed appointments, lack of discussion and feelings of being pressurised also fostered distrust. However, some parents trusted health professionals and more generally the NHS. Disclosure from health professionals regarding their own child's vaccination status facilitated vaccination and those with a friend who worked as a health professional felt a deeper level of reassurance.

Information presented in the media attenuated parents’ trust in key vaccination stakeholders and often dissuaded them from vaccinating. Parents in some articles had an attentional bias towards negative information, dismissing scientific information.

#### 3.3.6 Theme 9: Practical issues influence vaccination receipt post-decision

Practical issues made vaccination difficult for parents who decided to obtain a vaccination for their child. Difficulties included: travelling to the clinic, arranging childcare for other children during the vaccination appointment, not receiving reminders about appointments, lack of time (particularly for mothers who had returned to work), and practical features of general practice (for example, being unable to get an appointment). For other parents, having sufficient time to vaccinate and practical steps taken by healthcare providers facilitated vaccination.

## 4 Discussion

This qualitative systematic review identified two distinct types of decision-making about vaccination among parents in the UK: non-deliberative and deliberative. Non-deliberative decisions were those in which parents were happy to comply, felt they did not have a choice or followed social norms. These decisions were characterised by being quick, and not involving an explicit weighing up of the pros and cons of vaccination. By contrast, parents making deliberative decisions weighed up the risks and benefits of vaccination, considered others’ advice/experiences and were affected by beliefs about social judgement. Parents making deliberative decisions were influenced by their emotions, in which the media also played a role. The review identified that trust was integral to non-deliberative and deliberative decisions, with trust in information and those offering vaccination influenced by portrayals of vaccinations in the media. Practical issues affected some parents who intended to vaccinate their children.

Kahneman's Two Systems approach and Fuzzy Trace Theory (separate, but closely aligned approaches) also suggest that individuals’ decision-making occurs in two similar ways (Kahneman, 2003; Reyna, 2012). Individuals make effortful and conscious decisions (similar to deliberative decision-making), as well as automatic or gist-based decisions (akin to non-deliberative decision-making). In this review, some parents were seen to adopt both deliberative and non-deliberative decision-making at different times, suggesting that decision-making does not fall cleanly into an effortful/conscious approach versus an automatic approach. Use of each approach might be modulated by how familiar each vaccine context feels to parents (for example, does a vaccine ‘feel’ like a routine one, or is there something different for parents to consider?). Automatic decisions are driven in part by emotions (Kahneman, 2003), although this was not evident in the present review. Heuristics (or cognitive shortcuts) are used in automatic or gist-based decision-making and have helped us to understand that decision-making is affected by how messages about vaccination are presented (or ‘framed’). Individuals have a preference for avoiding losses (e.g. mild vaccine side-effects) over gains (e.g. disease protection) for frequent behaviours, but a preference for the reverse for one-off behaviours and this was reflected in the present review (Gerend, Shepherd, & Monday, 2008; Kahneman & Tversky, 1979; Rothman & Salovey, 1997). However, determining whether and/or under what circumstances framing increases vaccination uptake may be complex. A recent review of interventions to increase intentions to receive HPV vaccination found no study to report a main effect of gain versus loss framing, but interaction effects were reported (Fu, Bonhomme, Cooper, Joseph, & Zimet, 2014). In our review most data referred to parents making deliberative decisions, which may be explained by the fact that the majority of articles were published after the publication of a now retracted article in the Lancet in 1998 that linked MMR to autism and bowel disease (Godlee, Smith, & Marcovitch, 2011). This may have biased our results towards a focus on deliberative decision-making.

While there has been a move from encouraging patients to unquestioningly comply with health professionals, towards making informed decisions (Hargreaves et al., 2005, Raffle, 2001, Rimer et al., 2004), evidence suggests that conscious/effortful thinking might not result in good decision-making (Kahneman, 2003) and our review suggests that some people find this difficult. Furthermore, some parents are happy to go along with the recommendations of vaccination experts without considering the decision further and we know that the use of ‘presumptive’ communication (for example, ‘your child is due for the HPV vaccine’) is associated with greater vaccine acceptance compared with ‘participatory’ communication (for example, ‘what do you want to do about the HPV vaccine?’) (Opel, Mangione-Smith, Robinson, Heritage, DeVere, & Salas, 2015). Presumptive communication may shift parents into making a non-deliberative decision, which although it may increase vaccine uptake, may not be the best way to promote informed decision-making (Opel et al., 2015). The ‘consider an offer’ approach, put forward to facilitate patients making decisions about attending screening, might suit parents’ needs better (Entwistle, Carter, & Trevena, 2008). In this approach, communicators would recommend vaccination, discuss why it is being offered, help parents assess the appropriateness of vaccination for their child and provide additional information where needed. Parents can then respond to the recommendation in a manner that suits them; some may accept the recommendation from a health professional, while others may want further discussion. There may be a need for interventions to facilitate this discussion, based on the findings of this review, so that health professionals can anticipate and appropriately respond to parents’ queries. Such interventions need to be developed in collaborative partnership between parents, policy makers and health professionals. The ‘consider an offer’ approach will work best in settings involving parents and individual health professionals (rather than community / school-based programmes). It must also be acknowledged that health professionals will not always be a trusted source of advice and, as suggested in our review, parents might defer to the media or other parents.

The findings of this review provide an understanding of the factors underlying parents’ vaccination behaviour and highlight targets that will help us to better design interventions to enhance informed uptake. Of particular interest is the social aspect of vaccine decision-making. Many parents who discussed making non-deliberative decisions had opted to vaccinate their child, although some did so because they felt pressure to. However, others had copied other parents and had not vaccinated their child. Some parents had involved others in their deliberative decision-making. These findings highlight the importance of understanding vaccination decision-making at a social level; the impact of one child being unvaccinated may go beyond just that child being unprotected.

### 4.1 Limitations

This study had limitations, particularly in relation to our method. Study quality was assessed for the whole article, however some articles reported quantitative and qualitative findings so our assessment may not truly reflect the qualitative aspects of the studies. The review focuses on UK studies, and, while our findings might apply to other countries that have similar programmes, decision-making among parents in different contexts may differ (such as in countries where vaccines are not free-at-the-point-of-receipt and with different historical vaccination experiences, for example, parents in the UK were largely unexposed to the thimerosal scare in the USA about mercury content in multi-dose vials of vaccines). However, the social psychology of non-vaccination decisions is likely to be comparable between countries. Even within the UK, parents’ attitudes differ across vaccines and will vary by socio-demographic factors, which was not considered in this review. Relatedly, most articles focused on MMR immunisation, which limits the extent to which we can generalise our findings to other immunisations. The focus of papers on MMR in the UK is likely due to the publication of the 1998 Lancet paper, (Godlee et al., 2011) which resulted in a decrease in MMR uptake in the UK and has been followed by outbreaks of measles (Public Health Wales, 2014). All but one of the included studies were conducted after the publication of that paper, so our paper must be considered as an appraisal of vaccination decisions in this era. A difficulty with any review is that researchers do not have access to the raw data, so our interpretation is reliant on the original authors’ analyses and decisions about which quotes to report. Finally, our qualitative method does not allow us to determine the frequency of each type of decision-making at a population level.

### 4.2 Conclusions

Our review identified two very different approaches to decision-making about childhood vaccinations: deliberative and non-deliberative. Parents’ balancing of the risks and benefits of vaccination and their trust in immunisation providers are influential in their decision-making. Some parents express concern about social judgement of not immunising and some parents’ decisions are bespoke to their perceptions of their child's vulnerability to infection and vaccine side-effects. By understanding more about the mechanisms underlying parents’ vaccination behaviour, in collaborative partnership with vaccination stakeholders, we can better design interventions to enhance informed uptake.

## Conflicts of interest

None declared.

## Funding

This work was supported by Cancer Research UK [Grant numbers C49896/A17429 to AF, C7492/A17219 to JW, C42785/A17965 to SS]. The funder played no role in the study design, collection analysis or interpretation of data, in the writing of the article or the decision to submit for publication.

## Sources of support

AF and LR are funded by a Cancer Research UK – BUPA cancer prevention Fellowship awarded to AF (C49896/A17429). JW, LM and AC are funded by a Cancer Research UK Career Development Fellowship awarded to JW (C7492/A17219). HB is funded by the Higher Education Council for England. SS is funded by a Cancer Research UK post-doctoral Fellowship (C42785/A17965).

## Footnotes

Appendix A Supplementary data associated with this article can be found in the online version at doi:10.1016/j.ssmph.2016.07.005.
