# Supplementary material for: A qualitative systematic review of factors influencing parents’ vaccination decision-making in the United Kingdom
Source: SSM Popul Health. 2016 Aug 30;2:603–12. doi: 10.1016/j.ssmph.2016.07.005 (PMC5165048; doi:10.1016/j.ssmph.2016.07.005)
Supplement: Supplementary file 1 — Supplementary material [file mmc1.docx]

**SUPPLEMENTARY MATERIAL**

**Search terms by database**

|  | **PsycINFO** | **MEDLINE** | **Embase** | **Social Policy and Practice** | **CINAHL plus** | **Web of science** |
| --- | --- | --- | --- | --- | --- | --- |
| **Vaccination** | exp Immunization/  "vaccination*".ab,ti.  "immuni?ation*".ab,ti. | exp Immunization/  "vaccination*".ab,ti.  "immuni?ation*".ab,ti. | exp Immunization/  "vaccination*".ab,ti.  "immuni?ation*".ab,ti. | exp Immunization/  "vaccination*".ab,ti.  "immuni?ation*".ab,ti. | Exp Immunization/  “Immuni?ation*”  “Vaccination*” | “Immuni?ation*”  “Vaccination*” |
|  |  |  |  |  |  |  |
| **Qualitative** | exp Qualitative Research/  exp Interviews/  exp Observation Methods/  "interview*".ab,ti.  "focus group*".ab,ti.  "ethnogr*".ab,ti.  exp Ethnography/  “thematic analysis”.ab,ti.  “grounded theory”.ab,ti.  “interpretative phenomenological analysis”.ab,ti.  “content analysis”.ab,ti.  “framework analysis”.ab,ti. | exp Qualitative Research/  exp Interviews/  exp Observation Methods/  "interview*".ab,ti.  "focus group*".ab,ti.  "ethnogr*".ab,ti.  exp Ethnography/  “thematic analysis”.ab,ti.  “grounded theory”.ab,ti.  “interpretative phenomenological analysis”.ab,ti.  “content analysis”.ab,ti.  “framework analysis”.ab,ti. | exp Qualitative Research/  exp Interviews/  exp Observation Methods/  "interview*".ab,ti.  "focus group*".ab,ti.  "ethnogr*".ab,ti.  exp Ethnography/  “thematic analysis”.ab,ti.  “grounded theory”.ab,ti.  “interpretative phenomenological analysis”.ab,ti.  “content analysis”.ab,ti.  “framework analysis”.ab,ti. | exp Qualitative Research/  exp Interviews/  exp Observation Methods/  "interview*".ab,ti.  "focus group*".ab,ti.  "ethnogr*".ab,ti.  exp Ethnography/  “thematic analysis”.ab,ti.  “grounded theory”.ab,ti.  “interpretative phenomenological analysis”.ab,ti.  “content analysis”.ab,ti.  “framework analysis”.ab,ti. | exp Qualitative Studies/  exp Anthropology, Cultural/  exp Ethnographic Research/  exp Interviews/  exp Semi-structured Interview/  exp Structured Interview/  exp Focus Group/  exp Nonexperimental Studies/  exp Content Analysis/  exp Thematic Analysis/  exp Grounded Theory/  Qualitative*  Anthropology*  Ethnog*  Interview*  “Focus group”*  Observation*  “Content analysis”  “Thematic analysis”  “Grounded theory” | Qualitative*  Interview*  “Focus group”*  Observat*  Ethnog*Anthrop*  “Content analysis”  “Framework analysis” “Interpretative phenomenological analysis”  “Grounded theory” |
|  |  |  |  |  |  |  |
| **United Kingdom** | "United Kingdom".ab,ti.  "Great Britain".ab,ti.  England.ab,ti.  Scotland.ab,ti.  Wales.ab,ti.  "Northern Ireland".ab,ti. | "United Kingdom".ab,ti.  "Great Britain".ab,ti.  England.ab,ti.  Scotland.ab,ti.  Wales.ab,ti.  "Northern Ireland".ab,ti | "United Kingdom".ab,ti.  "Great Britain".ab,ti.  England.ab,ti.  Scotland.ab,ti.  Wales.ab,ti.  "Northern Ireland".ab,ti | "United Kingdom".ab,ti.  "Great Britain".ab,ti.  England.ab,ti.  Scotland.ab,ti.  Wales.ab,ti.  "Northern Ireland".ab,ti | United Kingdom/  England/  Great Britain/  Scotland/  Wales/  Northern Ireland/  “United Kingdom”  “Great Britain”  “England”  “Northern Ireland”  “Wales”  “Scotland” | “United Kingdom”  “Great Britain”  “England”  “Northern Ireland”  “Wales”  “Scotland” |

**PRISMA CHECKLIST**

| **Section/topic** | **#** | **Checklist item** | **Reported on page #** |
| --- | --- | --- | --- |
| **TITLE** | | |  |
| Title | 1 | Identify the report as a systematic review, meta-analysis, or both. | 1 |
| **ABSTRACT** | | |  |
| Structured summary | 2 | Provide a structured summary including, as applicable: background; objectives; data sources; study eligibility criteria, participants, and interventions; study appraisal and synthesis methods; results; limitations; conclusions and implications of key findings; systematic review registration number. | 2/3 |
| **INTRODUCTION** | | |  |
| Rationale | 3 | Describe the rationale for the review in the context of what is already known. | 4/5 |
| Objectives | 4 | Provide an explicit statement of questions being addressed with reference to participants, interventions, comparisons, outcomes, and study design (PICOS). | 5 |
| **METHODS** | | |  |
| Protocol and registration | 5 | Indicate if a review protocol exists, if and where it can be accessed (e.g., Web address), and, if available, provide registration information including registration number. | n/a |
| Eligibility criteria | 6 | Specify study characteristics (e.g., PICOS, length of follow-up) and report characteristics (e.g., years considered, language, publication status) used as criteria for eligibility, giving rationale. | 6 and supplementary material |
| Information sources | 7 | Describe all information sources (e.g., databases with dates of coverage, contact with study authors to identify additional studies) in the search and date last searched. | 6 |
| Search | 8 | Present full electronic search strategy for at least one database, including any limits used, such that it could be repeated. | Supplementary material |
| Study selection | 9 | State the process for selecting studies (i.e., screening, eligibility, included in systematic review, and, if applicable, included in the meta-analysis). | 6/7 |
| Data collection process | 10 | Describe method of data extraction from reports (e.g., piloted forms, independently, in duplicate) and any processes for obtaining and confirming data from investigators. | 6 |
| Data items | 11 | List and define all variables for which data were sought (e.g., PICOS, funding sources) and any assumptions and simplifications made. | 6 |
| Risk of bias in individual studies | 12 | Describe methods used for assessing risk of bias of individual studies (including specification of whether this was done at the study or outcome level), and how this information is to be used in any data synthesis. | 7 |
| Summary measures | 13 | State the principal summary measures (e.g., risk ratio, difference in means). | n/a |
| Synthesis of results | 14 | Describe the methods of handling data and combining results of studies, if done, including measures of consistency (e.g., I^2^) for each meta-analysis. | 7 |
| Risk of bias across studies | 15 | Specify any assessment of risk of bias that may affect the cumulative evidence (e.g., publication bias, selective reporting within studies). | 7 |
| Additional analyses | 16 | Describe methods of additional analyses (e.g., sensitivity or subgroup analyses, meta-regression), if done, indicating which were pre-specified. | n/a |
| **RESULTS** | | |  |
| Study selection | 17 | Give numbers of studies screened, assessed for eligibility, and included in the review, with reasons for exclusions at each stage, ideally with a flow diagram. | 8 and figure 1 |
| Study characteristics | 18 | For each study, present characteristics for which data were extracted (e.g., study size, PICOS, follow-up period) and provide the citations. | 8 and table 1 |
| Risk of bias within studies | 19 | Present data on risk of bias of each study and, if available, any outcome level assessment (see item 12). | Supplementary material |
| Results of individual studies | 20 | For all outcomes considered (benefits or harms), present, for each study: (a) simple summary data for each intervention group (b) effect estimates and confidence intervals, ideally with a forest plot. | n/a |
| Synthesis of results | 21 | Present results of each meta-analysis done, including confidence intervals and measures of consistency. | n/a |
| Risk of bias across studies | 22 | Present results of any assessment of risk of bias across studies (see Item 15). | 8 |
| Additional analysis | 23 | Give results of additional analyses, if done (e.g., sensitivity or subgroup analyses, meta-regression [see Item 16]). | n/a |
| **DISCUSSION** | | |  |
| Summary of evidence | 24 | Summarize the main findings including the strength of evidence for each main outcome; consider their relevance to key groups (e.g., healthcare providers, users, and policy makers). | 18 |
| Limitations | 25 | Discuss limitations at study and outcome level (e.g., risk of bias), and at review-level (e.g., incomplete retrieval of identified research, reporting bias). | 21 |
| Conclusions | 26 | Provide a general interpretation of the results in the context of other evidence, and implications for future research. | 18-22 |
| **FUNDING** | | |  |
| Funding | 27 | Describe sources of funding for the systematic review and other support (e.g., supply of data); role of funders for the systematic review. | Title page |

**References of articles included in the review**

Anderson ES, Jackson A, Wailoo MP, Petersen SA. Child care decisions: parental choice or chance? Child Care Health and Development 2002;28(5):391-401.

Austin H. Parents' perceptions of information on immunisations. Journal of Child Health Care 2001;5(2):54-59.

Austin H, Campion-Smith C, Thomas S, Ward W. Parents' difficulties with decisions about childhood immunisation. Community Practitioner 2008;81(10):32-35.

Brown KF, Long SJ, Ramsay M, Hudson MJ, Green J, Vincent CA, et al. U.K. parents' decision-making about measles-mumps-rubella (MMR) vaccine 10 years after the MMR-autism controversy: a qualitative analysis. Vaccine 2012;30(10):1855-1864.

Brownlie J, Howson A. 'Leaps of faith' and MMR: An empirical study of trust. Sociology-the Journal of the British Sociological Association 2005;39(2):221-239.

Casiday RE. Children's health and the social theory of risk: Insights from the British measles, mumps and rubella (MMR) controversy. Social Science and Medicine 2007;65(5):1059-1070.

Condon L. Maternal attitudes to preschool immunisations among ethnic minority groups. Health Education Journal 2002;61(2):180-189.

Cunningham CJ, Charlton CPJ, Jenkins SM. Immunization uptake and parental perceptions in a strictly orthodox Jewish community in north-east London. Journal of Public Health Medicine 1994;16(3):314-317.

Evans M, Stoddart H, Condon L, Freeman E, Grizzell M, Mullen R. Parents' perspectives on the MMR immunisation: a focus group study. British Journal of General Practice 2001;51(472):904-910.

Gardner B, Davies A, McAteer J, Michie S. Beliefs underlying UK parents' views towards MMR promotion interventions: a qualitative study. Psychology Health & Medicine 2010;15(2):220-230.

Gordon D, Waller J, Marlow LA. Attitudes to HPV vaccination among mothers in the British Jewish community: reasons for accepting or declining the vaccine. Vaccine 2011;29(43):7350-7356.

Guillaume LR, Bath PA. The impact of health scares on parents' information needs and preferred information sources: a case study of the MMR vaccine scare. Health Informatics Journal 2004;10(1):5-22.

Henderson L, Clements A, Damery S, Wilkinson C, Austoker J, Wilson S, et al. 'A false sense of security'? Understanding the role of the HPV vaccine on future cervical screening behaviour: a qualitative study of UK parents and girls of vaccination age. Journal of Medical Screening 2011;18(1):41-45.

Henderson L, Millett C, Thorogood N. Perceptions of childhood immunization in a minority community: qualitative study. Journal of the Royal Society of Medicine 2008;101(5):244-251.

Hill MC, Cox CL. Influencing factors in MMR immunisation decision making. British Journal of Nursing 2013;22(15):893-898.

Hilton S, Petticrew M, Hunt K. 'Combined vaccines are like a sudden onslaught to the body's immune system': parental concerns about vaccine 'overload' and 'immune-vulnerability'. Vaccine 2006a;24(20):4321-4327.

Hilton S, Hunt K, Petticrew M. Gaps in parental understandings and experiences of vaccine-preventable diseases: a qualitative study. Child: Care, Health & Development 2006b;33(2):170-179.

Hilton S, Petticrew M, Hunt K. Parents' champions vs. vested interests: Who do parents believe about MMR? A qualitative study. BMC Public Health 2007;7.

Johnson S, Capdevila R. ‘That’s just what’s expected of you … so you do it’: Mothers discussions around choice and the MMR vaccination. Psychology & Health 2014;29(8):861-876.

Kennedy C, Gray Brunton C, Hogg R. 'Just that little bit of doubt': Scottish parents', teenage girls' and health professionals' views of the MMR, H1N1 and HPV vaccines. International Journal of Behavioral Medicine 2014;21(1):3-10.

Lewendon GJ, Maconachie M. Why are children not being immunised? Barriers to immunisation uptake in South Devon. Health Education Journal 2002;61(3):212-220.

Marlow LA, Wardle J, Forster AS, Waller J. Ethnic differences in human papillomavirus awareness and vaccine acceptability. Journal of Epidemiology & Community Health 2009a;63(12):1010-1015.

Marlow LA, Wardle J, Waller J. Attitudes to HPV vaccination among ethnic minority mothers in the UK: an exploratory qualitative study. Human Vaccines 2009b;5(2):105-110.

McMurray R, Cheater FM, Weighall A, Nelson C, Schweiger M, Mukherjee S. Managing controversy through consultation: a qualitative study of communication and trust around MMR vaccination decisions. British Journal of General Practice 2004;54(504):520-525.

Mixer RE, Jamrozik K, Newsom D. Ethnicity as a correlate of the uptake of the first dose of mumps, measles and rubella vaccine. Journal of Epidemiology & Community Health 2007;61(9):797-801.

Petts J, Niemeyer S. Health risk communication and amplification: learning from the MMR vaccination controversy. Health, Risk & Society 2004;6(1):7-23.

Poltorak M, Leach M, Fairhead J, Cassell J. 'MMR talk' and vaccination choices: An ethnographic study in Brighton. Social Science & Medicine 2005;61(3):709-719.

Raithatha N, Holland R, Gerrard S, Harvey I. A qualitative investigation of vaccine risk perception amongst parents who immunize their children: a matter of public health concern. Journal of Public Health Medicine 2003;25(2):161-164.

Sampson R, Wong L, Macvicar R. Parental reasons for non-uptake of influenza vaccination in young at-risk groups: a qualitative study. The British journal of general practice : the journal of the Royal College of General Practitioners 2011;61(588):e386-391.

Smaibegovic MS, Laing GJ, Bedford H. Why do parents decide against immunization? The effect of health beliefs and health professionals. Child: Care, Health & Development 2003;29(4):303-311.

Sporton RK, Francis SA. Choosing not to immunize: are parents making informed decisions? Family Practice 2001;18(2):181-188.

Tickner S, Leman PJ, Woodcock A. 'It's just the normal thing to do': Exploring parental decision-making about the 'five-in-one' vaccine. Vaccine 2007;25(42):7399-7409.

Tickner S, Leman PJ, Woodcock A. Parents' views about pre-school immunization: an interview study in southern England. Child Care Health and Development 2010;36(2):190-197.

Tomlinson N, Redwood S. Health beliefs about preschool immunisations: an exploration of the views of Somali women resident in the UK. Diversity & Equality in Health & Care 2013;10(2):101-113.

**ADDITIONAL QUOTES FROM THE THEMES**

| **Theme 4: Weighing up the risks and benefits of vaccination**  “…while parents questioned the need for the vaccine, most preferred to err on the side of caution and opt for immunization against tetanus.” Hilton, author comment.  **Benefits of vaccinating**  Is it necessary to prevent disease?  “… mumps was often a source of humour, and participants often laughed while holding their breath, puffing out their cheeks, or (men) crossing their legs and clasping their hands over their groins as if in pain. Some queried the need for girls to receive the mumps vaccine as they perceived mumps to be a disease that affected boys.” Hilton, author comment.  “With all these diseases they’re just not heard of nowadays and that does kind of push your mind as to well what’s the point, you know?” Tickner, participant comment.  Is vaccination an effective way of doing this?  “I feel that if god wants her to get it [an illness] she will get it.” Henderson, participant comment.  “We chose not to … there are many different flu viruses and that the jab only protects against the “most likely.” Sampson, participant comment. |
| --- |
| **Risks of vaccinating**  How likely are these risks?  “The final thing that clinched it was just [name of partner] and his like, sensible everyday comment, not rooted in medical history that “Well do we know anybody who’s had an adverse reaction?” Because that is rooted in fact. Tangible fact that we can both hold on to. It’s not a scientific report that we can’t understand, it’s actual everyday living, and the answer to that was no. And that’s why, that was the point that really made me realise we were definitely going to go ahead [and vaccinate].” McMurray, participant comment.  Mechanisms of harm and individualised vulnerability  “I think all that happens is if we just keep vaccinating everything we, just our immune systems will just turn in, nut allergy, multiple sclerosis, Parkinson’s you know?” Brown, participant comment.  “If you spilt the contents of one of the [vaccine] syringes it would be a biohazard, you’d have to severely clear up the room.” Brown, participant comment.  “I just don’t want them to think they can go out there and have sex and they’re protected against everything, when they’re not in actual fact.” Marlow, participant comment. |

(Continued)

| **Theme 5: Others’ experiences and advice**  **Others’ experiences**  “A friend of mine who I worked with lost her baby erm at about eight weeks from meningitis, which was just awful. So I mean that may have been something that’s influenced me to make sure that they get the Hib and stuff ‘cos it was just so tragic.” Tickner, participant comment.  “there was a family (…) they had a perfectly normal child who received MMR at age 2 and he subsequently became blind, mentally retarded and deaf. And I think that’s a pretty bad reaction!” Henderson, participant comment.  **Others’ advice**  “Well, to be honest with you, had I not been, dare I use the word, alerted by friends, who said have you thought about your views on immunization? I thought it was compulsory until people told me it wasn't” Sporton, participant comment.  **Theme 6: Social judgement**  “I’d feel really uncomfortable having to go into hospital and think that there are people looking at me thinking, my God, why didn’t she get him vaccinated? Let her baby become ill and potentially die or whatever.” Brown, participant comment.  “In addition to protecting their own child, 13 parents referred to the importance of immunisation for the population, believing they had a social responsibility to protect children and eradicate diseases from society” Tickner, author comment.  “In a more religious section of the community it could be perceived as raising a subject that people don’t wish to speak about at that age, it could be perceived as making an assumption about the child that actually could disadvantage them, and certainly in the very religious community in terms of arranged marriages” Gordon, author comment.  **Theme 7: Emotions affecting decision-making**  “Both Somali and Afro-Caribbean women had seen television news items showing purportedly vaccine damaged children, which they found upsetting and worrying.” Condon, author comment  “...the diseases being immunised against, especially polio and meningitis, were particularly frightening.” Casiday, author comment.  “I think I cried more than they did. I felt really guilty, not because I was having them immunised, but because of the pain they were going to go through, terribly embarrassing.” Austin, participant comment.  “Although it might be a very, very small percentage risk, it’s your child and if it gets that, you have to deal with that for the rest of your life, I mean would you ever forgive yourself?” Brownlie, participant comment.  “Although parents recognized serious side effects to be rare, these often provoked feelings of ‘dread’”. Raithatha, author comment.  “I suppose if anything does happen to them and you had a choice to immunise them and you didn’t you could only blame yourself really.” Petts, participant comment. |
| --- |

(Continued)

| **Theme 8: Trust in vaccine information and vaccine stakeholders**  “…[the health professionals] gave me a lot of stuff which basically I couldn't understand most of it, it was all really medical obviously and a lot of it went over my head...” Sporton, participant comment.  “Although parents recognised that information in the media can be sensationalised, reassurances about the safety of the vaccine issued by the Department of Health were treated with scepticism as parents felt that their concerns had not been adequately addressed”. Evans, author comment  **Theme 9: Practical issues influence vaccination receipt post-decision**  “…she was just like, ‘Well I’m sorry, the nurse does jabs on Tuesday mornings and if you can’t make that, then there’s not really a lot I can do...” Tickner, participant comment.  “it doesn’t cost any money and why not?’” Henderson, participant comment.  “vaccinating at school had made it easy for them to get their daughter vaccinated” Gordon, author comment. |
| --- |

**Detailed description of themes 4 and 8**

**Theme 4: Weighing up the risks and benefits of vaccination**

The articles presented that parents engage in a process of weighing up the risks and benefits of (not) vaccinating. Although the discussion of the judgements parents made are laid out in a specific order below, they may happen in any sequence, or simultaneously. Most of the time, the decision to vaccinate was only made if the benefits were perceived to outweigh the risks. Although for some parents this was straightforward (vaccination is entirely beneficial/risky), for others, vaccination decisions were a “balancing act”. Some parents felt no level of risk was acceptable, while others chose to ‘hedge their bets’, taking the attitude that if the vaccine does no harm then they might as well get it.

“…while parents questioned the need for the vaccine, most preferred to err on the side of caution and opt for immunization against tetanus.” Hilton, author comment.

**Benefits of vaccinating.**

Is it necessary to prevent disease?

Parents considered whether their child having a disease was a negative thing that they wanted to avoid. This was based on whether a disease was potentially fatal, or could cause serious lifelong effects. This assessment was sometimes made in relation to other diseases or the sex of the child. Public health campaigns caused parents to perceive that diseases focused on in the campaign were more serious, but the absence of similar campaigns for other diseases was taken as a sign that these diseases are not a threat.

Parents also assessed whether they believed their child would be exposed to a disease. Many diseases were perceived to not be a particular threat within the UK and some believed that their lifestyles and environment protected their child sufficiently or provided reason to immunise.

“I suppose because I was at home with him, for the first, his first year of life, I knew that he wouldn’t be exposed to anything, he wasn’t going to a nursery or a child minder… I knew that to some extent I had some degree of control over the people he was exposed to and the germs he was exposed to.” Sporton, participant comment.

Diseases which were seen to be transmitted just through ‘being around someone’ were considered more important to protect against than those which are seen to have ‘behavioural’ modes of transmission such as sexual intercourse.

“Coming from a Muslim background… we don’t have sex before marriage for example, so your first experiences of these things are when you’re married and you stay in a relationship… because of that reason I’d probably say no, I wouldn’t bother with it with my two girls.” Marlow, participant comment.

Is vaccination an effective way of doing this?

Some parents expressed that they were also only likely to vaccinate their child if they believed that vaccination works. Knowledge of scientific reports, historical changes in disease prevalence, or a general trust in medicine informed parents’ assessments.

When parents doubted vaccination efficacy, they were concerned that vaccines do not cover all strains of viruses that cause a disease or believed that efficacy was short-lived. Some parents also believed that ‘naturally acquired’ immunity is superior to that gained through vaccination. Other parents believed that regardless of the efficacy of vaccination, it may be overridden by more powerful forces, such as God or fate.

“If children get measles, mumps, and rubella it helps build up their natural immunity, and that’s better than the immunity built up by vaccines.” McMurray, participant comment.

**Risks of vaccinating**

Parents took into account whether there were any potential serious (i.e. fatal, or life-altering) side-effects. Parents recognised that there were short-term, minor side effects of vaccination, but these were not sufficient to outweigh the benefits of vaccinating (although they did have emotional effects on the parent, discussed below).

One of the common concerns evoked was that the MMR may cause autism, or more general developmental problems, despite awareness that the study that raised this concern had been discredited. Some parents extrapolated this concern to other vaccines, whereas others evaluated the risk of other vaccines in relation to the ‘higher risk’ MMR, which made them more confident in those vaccination decisions.

“I know that the research that Andrew Wakefield has done and his findings. I know that it is not categorically proven but to me there is enough evidence to be questioned.” Guillaime, participant comment.

How likely are these risks?

Some parents who believed that vaccinations have the potential to cause side-effects assessed the level of risk to their own child, based on how common side-effects are in the general population using their experiential knowledge and instinct. Parents also considered their family history or their child’s past history of minor illnesses (e.g. colds and ear infections), chronic conditions or premature birth.

“the second one had lots of colds, he had allergies and eczema, and em, it just seemed to be too much on his wee immune system and I just felt it was too risky, whereas the third one is a much more robust child…” Hilton, participant comment.

Mechanisms of harm and individualised vulnerability

Parents conceptualised the mechanisms by which vaccines cause harm in three ways: 1) by weakening the immune system or sending it into ‘over-drive’ (particularly a concern when a child is ill); 2) vaccine ingredients causing harm; 3) vaccines causing an increase in high risk behaviour (relevant for viruses with a ‘behavioural’ mode of transmission).

**Theme 8: Trust in vaccine information and stakeholders affects non-deliberative and deliberative decisions**

**Trust in key stakeholders and the information they provide**

Lack of trust in government confused parents’ decision-making. The sense of distrust originated from various issues including historic health scares that remained in parents’ memories, believing that the government conceals information, lack transparency in the information they publish and questioning the validity of official statistics. Parents queried the government’s motives for promoting vaccination, suggesting that it is a cost-saving activity.

“Generally parents did not have confidence in statements issued by the government about the safety of MMR and analogies were made with the BSE crisis”. Evans, author comment.

Similarly, lack of trust in healthcare professionals was frequently discussed, particularly parents’ concern that GPs are financially rewarded for high uptake of vaccinations. These concerns were heightened for parents who had previous negative experiences. Specific issues arising in the GP consultation, such as rushed appointments, lack of discussion and feelings of being pressurised into vaccinating also fostered distrust.

“…you're meant to trust your doctor implicitly and yet … they're getting paid for having so many people vaccinated…, and you start thinking 'well... who's got my wee boy's best interests at heart" Hilton, participant comment.

Conversely, some parents also trusted health professionals and more generally the NHS, which was considered as distinct from the government. Disclosure from health professionals regarding their own child’s vaccination status was rated as important and those with a friend who worked as a health professional offered a deeper level of reassurance.

“When doctors … shared their own stories about making such decisions as a parent … parents were reassured that their concerns had been taken seriously.” Casiday, author comment.

Distrust in vaccination research and drug development was commonly mentioned. Parents saw their children as being used as “guinea pigs” and disliked the uncertainty of scientific research.

“It makes me slightly untrusting […] I think well how can they just say that and just, so confidently, you know, think the atom is the smallest thing until they split it open and then it’s not and they can just so quickly just change and I think that’s, that’s hard when you’re trusting these people with your child’s health.” Johnson, participant comment.

Provision of information assisted in the decision-making process. However, often articles reported that parents were dissatisfied with the information they received, particularly because of their distrust in the information source. Parents perceived the information to be unclear, unengaging, lacking in quantity and delivered through an inappropriate medium.

Media influence

Information presented in the media attenuated the trust that parents held for official bodies’ research and development of the vaccines. Parents in some articles had an attentional bias towards negative information, dismissing scientific information that could counter sensationalised media stories. In general, the media’s influence tended to put parents off vaccinating their children.

“Although parents recognised that information in the media can be sensationalised, reassurances about the safety of the vaccine issued by the Department of Health were treated with scepticism as parents felt that their concerns had not been adequately addressed”. Evans, author comment.

**INCLUSION AND EXCLUSION CRITERIA**

**Types of studies**

Primary research studies meeting the following criteria:

- reporting qualitative analysis of textual data (collected using focus groups, interviews, participant observation, free-text questionnaire responses);
- and indexed at any time in online databases and published in peer reviewed journals in English.

We excluded dissertation abstracts, book chapters, review articles and commentaries.

**Types of participants**

Parents or caregivers of children/adolescents living in the United Kingdom. Participants must have been making decisions about vaccinating a child (under 18 years old).
